# Reduced serum and skeletal muscle MOTS c levels in women with polycystic ovary syndrome are associated with mitochondrial dysfunction

**DOI:** 10.1038/s41598-026-39687-x

**Published:** 2026-02-12

**Authors:** Irem Sonmezoglu Kutuk, Senay Akin, Haydar Demirel, Sezcan Mumusoglu, Turkmen Ciftci, Bulent Okan Yildiz

**Affiliations:** 1 Department of Internal Medicine, Hacettepe University School of Medicine, Ankara, Turkey; 2 Division of Exercise and Sport Physiology, Faculty of Sport Sciences, Hacettepe University, Ankara, Turkey; 3 Center for Sport Sciences, Near East University, Nicosia, Cyprus; 4 Department of Obstetrics and Gynecology, Hacettepe University School of Medicine, Ankara, Turkey; 5 Department of Radiology, Hacettepe University School of Medicine, Ankara, Turkey; 6 Division of Endocrinology and Metabolism, Hacettepe University School of Medicine, Ankara, Turkey; 7 Division of Endocrinology and Metabolism, Department of Internal Medicine, Hacettepe University School of Medicine, Hacettepe, Ankara, 06100 Turkey

**Keywords:** MOTS-c, Polycystic ovary syndrome, Mitochondrial-derived peptides, Skeletal muscle, Insulin resistance, Mitochondrial dysfunction, Biochemistry, Biomarkers, Diseases, Endocrinology, Medical research, Physiology

## Abstract

Polycystic ovary syndrome (PCOS) is characterized by insulin resistance and metabolic dysfunction. Mitochondrial-derived peptides (MDPs), including MOTS-c, regulate glucose homeostasis and skeletal muscle metabolism. Whether MOTS-c expression is altered in PCOS across different physiological compartments remains unknown. The aim was to assess circulating and skeletal muscle MOTS-c levels in women with PCOS and to examine their associations with metabolic and hormonal parameters. Forty women with PCOS and 40 age- and BMI-matched healthy controls underwent clinical, biochemical, and hormonal phenotyping. Serum MOTS-c concentrations were quantified by ELISA. In a representative subgroup, skeletal muscle MOTS-c expression was assessed in vastus lateralis biopsy specimens using Western blotting. Women with PCOS exhibited lower circulating MOTS-c concentrations compared with controls (220.2 ± 147.6 pg/mL vs. 498.3 ± 224.4 pg/mL, *p* < 0.001). Skeletal muscle MOTS-c expression was also reduced in the PCOS group (74.2 ± 15.2 vs. 100.0 ± 8.5 arbitrary units; *p* = 0.005). Serum MOTS-c levels were inversely associated with total testosterone (*r* = − 0.224, *p* = 0.046) and total cholesterol (*r* = − 0.228, *p* = 0.044). Women with PCOS display reduced MOTS-c expression in both the circulation and skeletal muscle, suggesting reduced availability of this mitochondrial-derived peptide. Associations with hyperandrogenism and lipid profiles suggest a potential link between altered mitochondrial peptide biology and the endocrine–metabolic phenotype of PCOS. These findings suggest that MOTS-c may represent a potential marker of tissue-specific mitochondrial involvement in PCOS and warrant further investigation.

**Supplementary Information:**

The online version contains supplementary material available at https://doi.org/10.1038/s41598-026-39687-x.

## Introduction

Polycystic ovary syndrome (PCOS) represents one of the most prevalent endocrine disorders among women of reproductive age, with a global prevalence estimated to range between 6% and 10%, depending on diagnostic criteria applied^1,2^. As defined by the Rotterdam criteria^3^, PCOS is characterized by chronic anovulation, hyperandrogenism, and polycystic ovarian morphology. Increasingly, the syndrome is recognized not merely as a reproductive disorder but as a complex, multisystem condition with significant metabolic implications. Affected women frequently exhibit insulin resistance (IR), visceral adiposity, dyslipidemia, and an elevated risk of developing type 2 diabetes mellitus (T2DM) and cardiovascular disease^4,5^. Although the precise etiology of PCOS remains incompletely elucidated, accumulating evidence implicates mitochondrial dysfunction as a key contributor to its pathophysiology^6^.

Mitochondria-derived peptides (MDPs) have recently been identified as important signaling molecules that modulate mitochondrial activity and contribute to the regulation of cellular and systemic metabolic homeostasis. One such peptide, MOTS-c (mitochondrial open reading frame of the 12 S rRNA-c), is a 16-amino acid peptide encoded within the 12 S rRNA region of mitochondrial DNA^7^. MOTS-c is predominantly expressed in metabolically active tissues, such as skeletal muscle, and is also detectable in the systemic circulation^8^. It regulates nuclear gene expression in response to cellular stress and promotes metabolic homeostasis through activation the folate – AICAR (5-aminoimidazole-4-carboxamide ribonucleotide) – AMPK (AMP-activated protein kinase) signaling pathway. This activation enhances glucose uptake, increases fatty acid oxidation, and modulates inflammatory responses^7^. Preclinical studies have demonstrated that exogenous MOTS-c administration improves insulin sensitivity, prevents diet-induced obesity, and enhances physical performance in animal models^7,8^.

Despite growing scientific interest, research on MOTS-c in humans, particularly in the context of endocrine and metabolic disorders such as PCOS, remains limited. Existing studies have reported reduced circulating MOTS-c levels in conditions including aging, obesity, and T2DM; however, data regarding its tissue-specific expression are sparse^9,10^. This knowledge gap is particularly pertinent to skeletal muscle, which functions both as a primary target and a major source of MOTS-c^7^. In addition to circulating MOTS-c levels, evaluating its expression in skeletal muscle, a tissue highly enriched in mitochondria, may provide novel insights into the mechanisms driving metabolic dysregulation in PCOS.

In a previous study, we examined another MDP, humanin, and observed significantly reduced serum levels along with unaltered skeletal muscle levels in women with PCOS^11^. These findings suggest that mitochondrial peptides may be differentially regulated across tissues in PCOS^11^. Whether MOTS-c exhibits a similar or distinct tissue-specific expression profile in this context remains to be determined.

The aim of the present study was to evaluate MOTS-c levels in both serum and skeletal muscle tissue of women with PCOS compared with healthy controls. By assessing both systemic and tissue-specific expression, this study seeks to determine whether MOTS-c may serve as a biomarker or play a mechanistic role in the metabolic disturbances associated with PCOS.

## Materials and methods

### Study population

This study was conducted between January and December 2023 at the outpatient Endocrinology and Metabolism clinic of Hacettepe University. A total of forty women diagnosed with PCOS according to the Rotterdam criteria^3^ were included. The diagnosis required the presence of at least two of the following: hyperandrogenism (HA), oligo/anovulation (OA), or polycystic ovarian morphology (PCOM), as defined previously^12^.

An age- and body mass index (BMI)-matched control group was composed of healthy women with regular menstrual cycles and without clinical or biochemical signs of hyperandrogenism or ultrasonographic evidence of PCOM. All participants were cisgender females; sex was defined based on biological and clinical criteria according to SAGER guidelines. All participants were required to complete the International Physical Activity Questionnaire (IPAQ) and only those classified as inactive or minimally active were enrolled to minimize the confounding effects of exercise.

Participants were excluded if they had a BMI less than 25 or greater than 40 kg/m², had experienced a body weight change exceeding 10% in the previous six months, had pre-existing diabetes or prediabetes, were smokers, were currently pregnant, had a history of chronic systemic illness, or had used any medications within the past three months. Women using hormonal contraception or other exogenous sex steroids were excluded.

The study was approved by the Ethics Committee of Hacettepe University (Approval number: KA-22046), and written informed consent was obtained from all participants in accordance with institutional guidelines and the Declaration of Helsinki.

### Clinical protocol

A comprehensive clinical evaluation was performed for all participants. Standardized forms were used to document medical history and anthropometric measurements, including height, weight, waist, and hip circumferences. Waist circumference was measured at the midpoint between the lower costal margin and the iliac crest, while hip circumference was measured at the level of the greater trochanters. BMI was calculated using the formula: weight (kg) divided by height squared (m^2^).

Body composition, including total fat mass and regional fat distribution, was assessed using segmental bioelectrical impedance analysis (BIA) with the TANITA BC-418 MA device (Tokyo, Japan). Hirsutism was evaluated using the modified Ferriman–Gallwey (mFG) scoring system^13,14^.

Blood samples were obtained during early follicular phase (days 2–5 of spontaneous or progesterone-induced menstrual bleeding) between 8:00 and 10:00 AM, following an overnight fast. A standard 75-g oral glucose tolerance test (OGTT) was performed in all subjects for assessment of glucose metabolism.

### Human biopsy acquisition

Skeletal muscle biopsy was performed in a subset of the study population, including 6 women with PCOS and 6 age- and BMI-matched healthy control subjects who voluntarily consented to undergo biopsy. All biopsies were performed under standardized pre-procedural conditions following an overnight fast, and participants were instructed to avoid activities that could acutely influence metabolic or mitochondrial measurements prior to the procedure. The procedure was performed under sterile conditions and local anesthesia using 2% prilocaine. A guillotine-type automatic 16-gauge biopsy needle (microbiopsy) was used under ultrasound guidance to obtain samples from the vastus lateralis muscle, following a modified Bergström technique^15^. Immediately after collection, tissue specimens were snap-frozen in liquid nitrogen and stored at − 80 °C until Western Blot analysis.

### Laboratory measurements

#### Biochemical and hormonal analyses

Triglycerides (TG), low density lipoprotein cholesterol (LDL-C), and high density lipoprotein cholesterol (HDL-C) concentrations were measured using enzymatic colorimetric kits with intra- and inter-assay coefficients of variation < 10%, plasma glucose concentration was determined by the glucose oxidase method (Olympus AU 2700; Beckman Coulter, Inc., Pasadena, CA) and serum total testosterone levels were measured by the chemiluminescent immunoassay (Roche Diagnostics GmbH, Mannheim, Germany), with intra- and inter-assay coefficients of variation both < 5%. Serum SHBG levels were measured by a two-step immunoenzymatic sandwich method (Beckman Coulter Inc., USA), with intra- and inter-assay coefficients of variation of 4.0% and 6.3%, respectively, and plasma insulin concentrations were measured using a single-step immunoenzymatic method (Beckman Coulter Inc., USA), with intra- and inter-assay coefficients of variation of 4.5% and 5.6%^16,17^. The Free Androgen Index (FAI) was calculated using the following formula: FAI = [Total Testosterone (nmol/L) / SHBG (nmol/L)] × 100. All biochemical and hormonal measurements were performed using validated automated systems under routine laboratory quality control conditions.

#### Serum MOTS-c measurements

To determine circulating MOTS-c levels, 10 mL of venous blood was collected from each participant into serum separator tubes (BD Vacutainer^®^ SST™ II Advance). Samples were centrifuged at 3,500 x g for 15 min at 4 °C, and the resulting serum was aliquoted and stored at − 80 °C until analysis.

Serum MOTS-c concentrations were quantified using a commercially available enzyme-linked immunosorbent assay (ELISA) kit (BT Lab, Cat# E6880Hu), according to the manufacturer's instructions. According to the manufacturer's specifications, the sensitivity of the assay was 4.08 pg/mL, with a detection range of 7–1500 pg/mL.

### Protein extraction and Western blot analysis

Frozen skeletal muscle biopsy samples were homogenized in radioimmunoprecipitation assay (RIPA) buffer supplemented with a Halt™ protease inhibitor cocktail (Thermo Fisher Scientific). The homogenates were centrifuged at 13,500 rpm for 15 min at 4 °C, and the resulting supernatant was stored at -80 °C for subsequent analysis.

Protein concentrations in the supernatants were determined spectrophotometrically using the BCA Protein Assay (Takara Bio Inc). Equal amounts of 40 µg of protein were loaded onto a 4–12% NuPAGE Bis-Tris gel (Invitrogen) and separated by electrophoresis in MES SDS running buffer. Proteins were then transferred onto 0.22 μm PVDF membranes (Thermo Scientific) using the Trans-Blot Turbo Transfer System (Bio-Rad). Membranes were blocked with Tris-buffered saline containing 0.1% Tween-20 (TBST) and 5% bovine serum albumin (BSA), followed by overnight incubation at 4 °C with a primary antibody against human MOTS-c (Fabgennix Cat# MOTSC-101AP, RRID: AB_3083067) diluted of 1:500. The MOTS-c antibody used in this study has been previously validated for the detection of human MOTS-c^18,19^, and a distinct immunoreactive band was consistently detected within the expected low–molecular weight range (~ 6 kDa) for MOTS-c. After washing with TBST, membranes were incubated for 1 h at room temperature with a horseradish peroxidase (HRP)-conjugated secondary antibody (Cell Signaling Technology Cat# 7074, RRID: AB_2099233). Protein bands were visualized using a chemiluminescence imaging system (Chemi-Doc, Bio-Rad) with Clarity Western ECL blotting substrate (Bio-Rad). Band intensities were quantified using ImageJ software and normalized to total protein levels as determined by Ponceau S staining.

For immunoblotting, proteins were separated by SDS–PAGE and transferred to PVDF membranes. Membranes were cut horizontally prior to antibody incubation to allow probing of proteins at different molecular weights. Blots were imaged using the same acquisition settings within each experiment. Cropping was performed only for figure presentation. No image manipulation beyond uniform adjustment of brightness and contrast was applied.

### Statistical analysis

Participants in the PCOS and control groups were matched for age and BMI using propensity score matching to reduce the impact of potential confounders on MOTS-c levels. Matching was performed in R statistical software (version 4.3.0) with the MatchIt, lmtest, and sandwich packages.

Categorical variables were reported as frequencies and percentages, while continuous variables were summarized using means and standard deviations or medians and interquartile ranges, depending on the distribution of the data. Normality was assessed using the Kolmogorov–Smirnov test.

Group comparisons for continuous variables were carried out using either the Student’s t-test or the Mann–Whitney U test, based on the normality of the data. Associations between categorical variables were evaluated using the chi-square test. Correlation analyses were conducted using Pearson’s or Spearman’s correlation coefficients, as appropriate. In addition to correlation analyses, multivariable linear regression models were constructed to assess independent associations between serum MOTS-c levels and metabolic and hormonal parameters, adjusting for potential confounders including age and BMI.

All statistical tests were two-sided, and a p-value of less than 0.05 was considered statistically significant. Analyses were performed using IBM SPSS Statistics for Windows, version 26.0 (IBM Corp., Armonk, NY, USA).

## Results

### Clinical, anthropometric, and biochemical characteristics

Among women with PCOS, phenotype A (HA + OA + PCOM) was the most common, observed in 77.5% of participants. Phenotype B (HA + OA) and phenotype C (HA + PCOM) were each present in 5%, while phenotype D (OA + PCOM) was observed in 12.5%, reflecting a predominance of the classical phenotype in the study group. The clinical and biochemical features of the study population are presented in Tables 1 and 2.

**Table 1:** Clinical and anthropometric characteristics of the study groups.

Variable	PCOS *N* = 40	Control *N* = 40	*P* Value
Age	21.8 (±2.3)	22.5 (±1.6)	0.14
Body weight (kg)	66.0 (±12.3)	62.2 (±9.1)	0.13
BMI (kg/m^2^)	24.4 (21.2–28.4)	22.9 (20.4–24.7)	0.19
mFG score	8 (5–10)	1 (0–2)	< 0.001
Waist (cm)	72.4 (±8.9)	65.6 (±4.7)	< 0.001
Waist/Hip Ratio (%)	0.71 (±0.5)	0.67 (±0.4)	< 0.001
Total body fat (kg)	21.0 (±9.1)	18.5 (±6.3)	0.16
Total body fat (%)	30.4 (±8.4)	28.9 (±5.9)	0.36

Data are given as mean (SD) and median (IQR) where appropriate. mFG score: modified Ferriman-Gallwey score.

**Table 2:** Hormonal and metabolic parameters in the study groups.

Variable	PCOS *N* = 40	Control *N* = 40	*P* Value
Total testosterone (ng/dL)	54.2 (40-62.2)	26.2 (21.0-32.9)	< 0.001
SHBG (nmol/l)	45.7 (28.8–62.7)	52.6 (43.6–75.0)	0.028
FAI	3.7 (2.9–5.5)	1.7 (1.2–2.2)	< 0.001
Fasting glucose (mg/dL)	84.9 (±8.8)	83.7 (±5.4)	0.49
Fasting insulin (µIU/mL)	7.0 (5.6–10.8)	6.2 (4.8–8.6)	0.71
2-hour glucose (mg/dL)	89.8 (±18.3)	82.7 (±14.7)	0.59
2-hour insulin (mg/dL)	33.63 (22.5–66.8)	25.1 (14.1–41.0)	0.01
Total cholesterol (mg/dL)	180.3 (±33.2)	165.8 (±32.4)	0.052
HDL-C (mg/dL)	59.4 (±12.1)	56.5 (±7.5)	0.207
LDL-C (mg/dL)	112.9 (±25.9)	99.4 (±23.3)	0.017
Triglyceride (mg/dL)	70 (52–103)	56 (42–75)	0.006

Data are given as mean (SD) and median (IQR) where appropriate.

SHBG: Sex hormone binding globulin, FAI: free androgen index, HDL: High density lipoprotein cholesterol, LDL: Low density lipoprotein cholesterol.

Women with PCOS had significantly higher fasting and 2-hour insulin levels during OGTT, and HOMA-IR values (*p* < 0.001 for all). Total testosterone and free androgen index (FAI) were also significantly elevated in the PCOS group (*p* < 0.001 for both). Additionally, LDL cholesterol and triglyceride levels were significantly higher in the PCOS group (*p* = 0.017, and *p* = 0.006 respectively).

### Serum MOTS-c levels

**Fig. 1:**
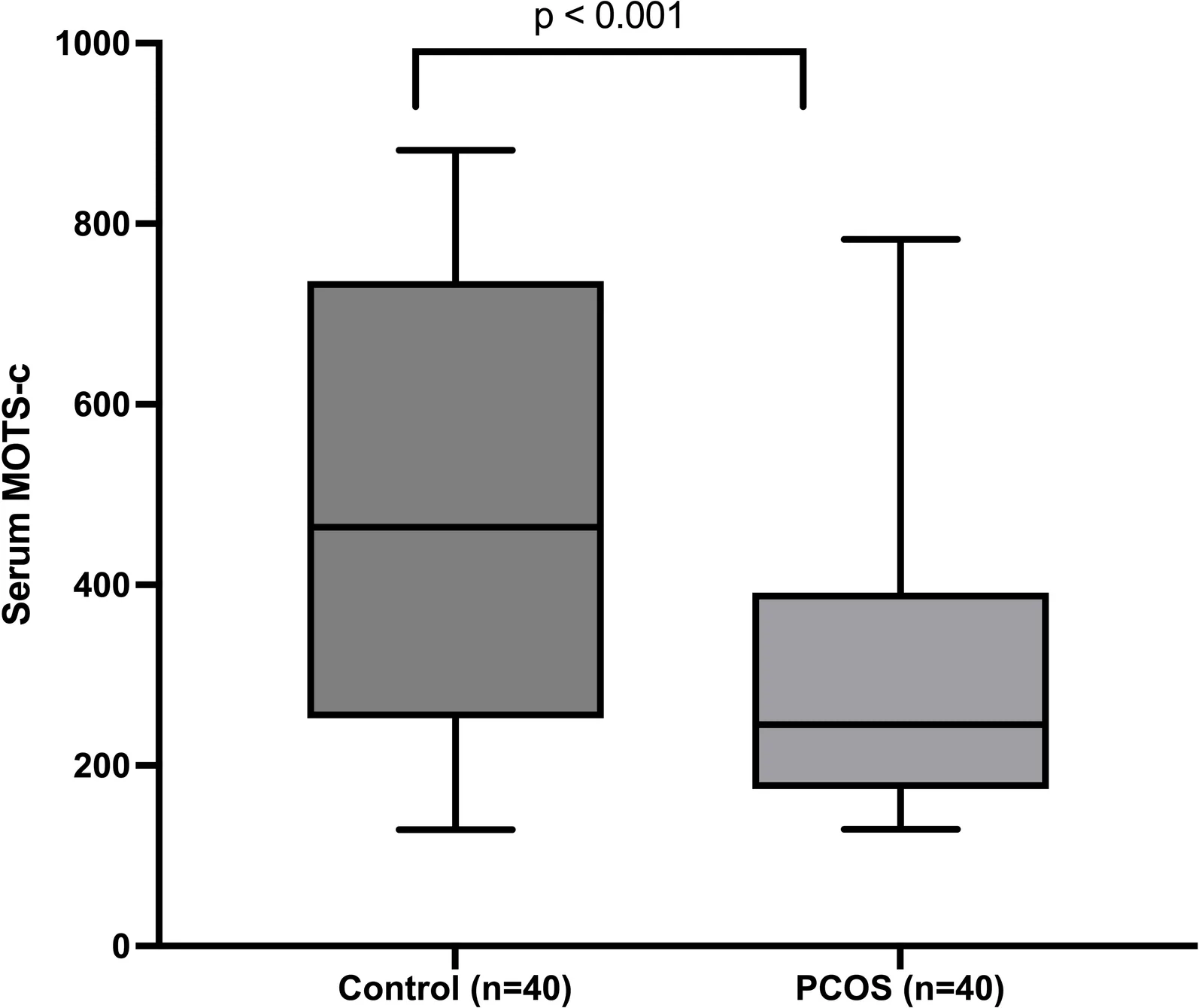
Serum MOTS-c levels in PCOS and control groups.

Serum MOTS-c concentrations were significantly lower in women with PCOS compared to healthy controls (220.2 ± 147.6 pg/mL vs. 498.3 ± 224.4 pg/mL, *p* < 0.001) (Fig. 1).

### Skeletal muscle MOTS-c expression analysis

**Fig. 2:**
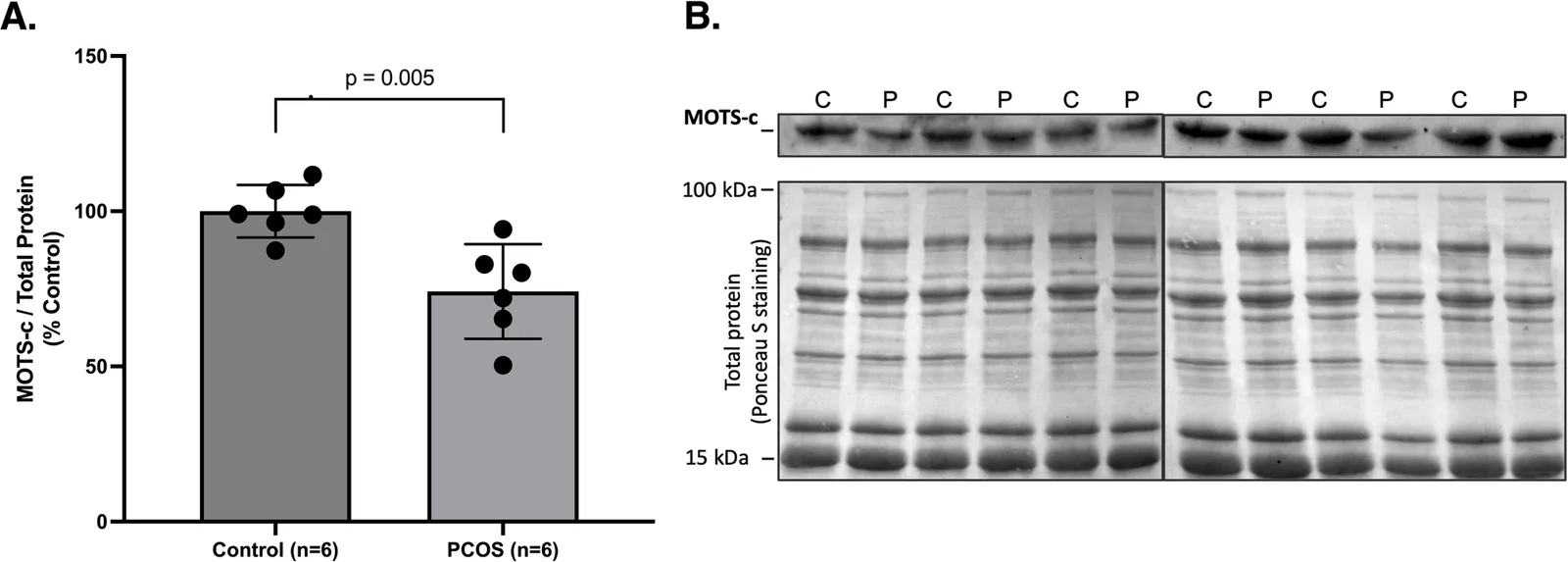
Skeletal muscle MOTS-c levels in control and PCOS groups (**A**) and Western blot images showing MOTS-c expression in skeletal muscle from control (C, *n* = 6) and PCOS (P, *n* = 6) subjects (**B**).

Skeletal muscle MOTS-c expression was evaluated in a biopsy subgroup consisting of 6 women with PCOS and 6 age- and BMI-matched healthy controls. Skeletal muscle MOTS-c levels were significantly lower in women with PCOS compared to healthy controls (74.2 ± 15.2 vs. 100.0 ± 8.5; *p* = 0.005), as shown in Fig. 2A. Western blot analysis supported these findings, demonstrating reduced MOTS-c expression in muscle biopsies of the PCOS group relative to controls (Fig. 2B).

### Correlation analyses

**Fig. 3:**
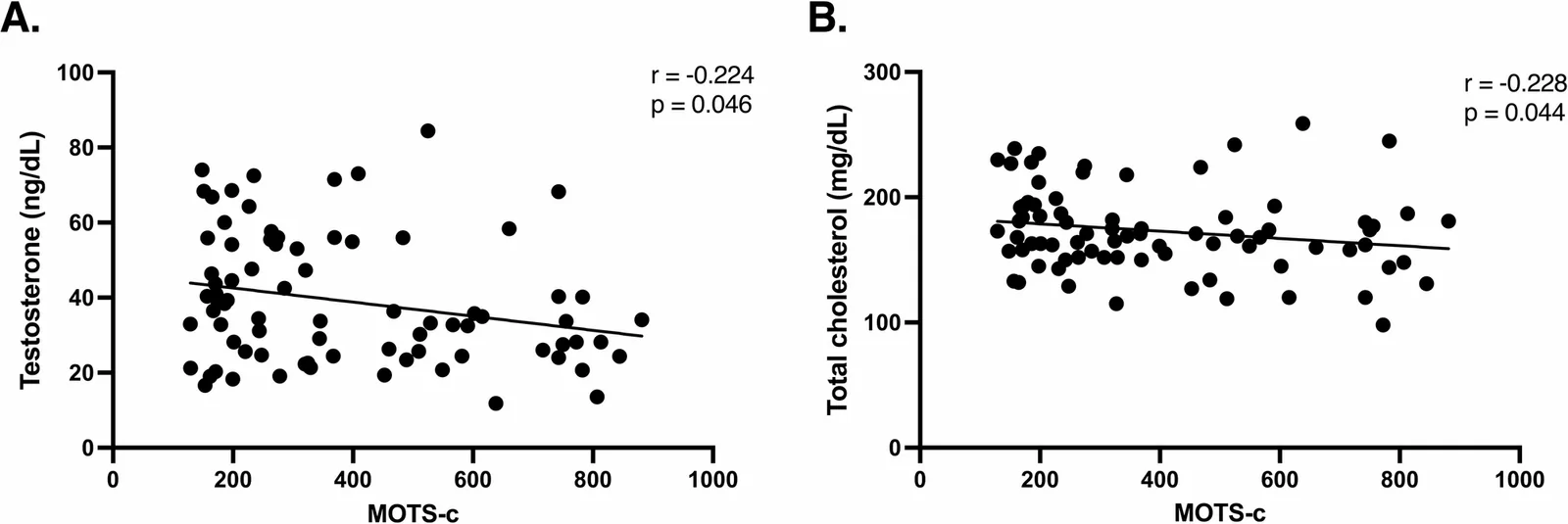
Correlations of serum MOTS-c with total testosterone (**A**) and total cholesterol (**B**). Correlations were assessed using Spearman’s correlation analysis.

There were significant negative associations between serum MOTS-c levels and total testosterone (*r* = – 0.224, *p* = 0.046) and total cholesterol (*r* = -0.228, *p* = 0.044) (Fig. 3). Serum MOTS-c levels showed a positive correlation with physical activity scores (*r* = 0.350, *p* = 0.001). Additionally, trends towards inverse correlations were observed with 2-hour insulin levels (*r* = -0.218, *p* = 0.052), triglycerides (*r* = -0.204, *p* = 0.057), and LDL cholesterol (*r* = -0.215, *p* = 0.052). All correlation analyses were performed in the combined cohort including both women with PCOS and healthy controls. In multivariable linear regression analyses adjusted for age and BMI, total testosterone and total cholesterol remained independently and inversely associated with serum MOTS-c levels. In models additionally including group status and physical activity, PCOS status and physical activity remained significant independent predictors of serum MOTS-c levels (Supplementary Tables S2–S5).

## Discussion

In our study, women with PCOS had significantly lower MOTS-c levels both in serum and in skeletal muscle tissue compared to healthy controls. This finding suggests that the reduction in MOTS-c is not confined to the circulation but may also reflect diminished expression within skeletal muscle. Furthermore, serum MOTS-c levels were negatively correlated with total testosterone and total cholesterol; however, these associations were weak in magnitude despite being statistically significant. These findings suggest an association between reduced MOTS-c levels and certain metabolic features of PCOS, although the biological relevance of these correlations remains to be determined. Notably, in multivariable linear regression models adjusting for age and BMI, both total testosterone and total cholesterol remained independently and inversely associated with serum MOTS-c levels. Overall, these results suggest that reduced MOTS-c levels are associated with metabolic disturbances characteristic of PCOS at both systemic and tissue levels.

The only human study examining MOTS-c in women with PCOS, conducted by Ramanjaneya et al., reported numerically lower baseline serum MOTS-c levels in the PCOS group compared with controls; however, this difference was not statistically significant, likely due to the small sample size (12 patients and 10 controls) and the study’s focus on lipid and insulin infusion rather than basal physiology^20^. Our study demonstrates a significant reduction in basal MOTS-c levels in both serum and skeletal muscle, suggesting that diminished MOTS-c may reflect metabolic alterations associated with PCOS.

Beyond PCOS, decreased MOTS-c levels have also been reported in individuals with T2DM, gestational diabetes mellitus, and obesity^7,21–23^. These studies collectively support the role of MOTS-c as a regulator of insulin sensitivity and glucose metabolism. For instance, Lee et al. demonstrated that lower circulating MOTS-c levels were associated with insulin resistance and inflammation in individuals with obesity^7^, while Kong et al. showed that MOTS-c delays the diabetes progression by preventing pancreatic β-cell senescence^23^. Mechanistically, MOTS-c activates AMP-activated protein kinase (AMPK), a key metabolic sensor that enhances glucose uptake, promotes fatty acid oxidation, and suppresses inflammatory signaling^7,24,25^. These pathways are highly relevant to the metabolic abnormalities characteristic of PCOS and may underlie the observed associations between reduced MOTS-c levels and insulin resistance in our study.

This is the first study to examine skeletal muscle MOTS-c expression in women with PCOS. Previous research has largely focused on circulating MOTS-c levels and has shown that skeletal muscle MOTS-c responds to exercise, aging, and various metabolic interventions^8,26^. However, these findings offer limited insight into MOTS-c biology in PCOS, as no previous studies have examined its expression directly in the skeletal muscle of this population. In our study, MOTS-c expression in the vastus lateralis was significantly lower in women with PCOS and its associations with metabolic parameters suggest that reduced muscle MOTS-c may reflect underlying disturbances such as insulin resistance, inflammation, and hormonal imbalance. Nevertheless, how MOTS-c responds to dynamic metabolic challenges in PCOS remains unknown and warrants further investigation.

In our previous study, we reported significantly decreased circulating humanin levels in women with PCOS compared with healthy controls, indicating a systemic mitochondrial stress response accompanied by reduced cytoprotective signaling^11^. The present study extends these observations by investigating another mitochondrial-derived peptide, MOTS-c, and demonstrates that its levels are reduced not only in serum but also in skeletal muscle tissue. Interestingly, muscle humanin levels remained unchanged, suggesting that these two MDPs, despite their shared origin, may be differentially regulated in response to the metabolic disturbances characteristic of PCOS.

Humanin is a broadly expressed, anti-apoptotic peptide that exerts cytoprotective effects across multiple tissues, often fuctioning as a generalized stress-response factor^27^. In contrast, MOTS-c is predominantly expressed in skeletal muscle and act as a metabolic regulator, promoting glucose uptake, fatty acid oxidation, and mitochondrial biogenesis through activation of the AMPK and CK2–AKT pathways^7,8,28^. Previous studies have shown that MOTS-c levels are highly responsive to metabolic stress, insulin resistance, and physical inactivity, whereas humanin expression tends to be more stable due to its widerspread tissue distribution and potential compensatory mechanisms^8,26,29^. Therefore, the selective reduction of muscle MOTS-c in PCOS may reflect impaired mitochondrial signaling and diminished “exercise-mimetic” capacity of skeletal muscle, referring to the ability of MOTS-c to activate key metabolic pathways such as AMPK and promote glucose uptake and metabolic flexibility in a manner similar to physical exercise, a hallmark of metabolic inflexibility in this condition. Conversely, the preservation of intramuscular humanin levels may represent a local compensatory mechanism that maintains basal cytoprotective responses despite systemic mitochondrial dysfunction. Collectively, these findings suggest that MOTS-c, rather than humanin, may serve as a more specific biomarker of muscle-derived mitochondrial stress and metabolic impairment in PCOS. Although all participants were classified as inactive or minimally active according to IPAQ scores, serum MOTS-c levels showed a positive correlation with physical activity scores. Notably, this association was observed even within a low-activity range, suggesting that MOTS-c may be sensitive to subtle differences in habitual physical activity rather than reflecting an exercise intervention effect.

One of the primary mechanisms through which MOTS-c exerts its metabolic actions is activation of the AMPK pathway, a central regulator of energy homeostasis. Activation of AMPK enhances glucose uptake, promotes fatty acid oxidation, stimulates mitochondrial biogenesis via the PGC-1α axis, and attenuates inflammation, all processes essential for maintaining insulin sensitivity and metabolic flexibility^7,30^. Moreover, MOTS-c has been shown to support oxidative muscle fiber composition and preserve pancreatic β-cell function, both critical for glucose regulation^23,31^. In PCOS, these pathways are often dysregulated. Chronic low-grade inflammation, insulin resistance, and hyperandrogenism impair AMPK signaling, leading to reduced mitochondrial function and energy dysregulation^6,32^. Thus, the observed decreases in both serum and skeletal muscle MOTS-c levels are consistent with a reduced activation of these protective metabolic pathways in PCOS. Impaired mitokine signaling could therefore be associated with insulin resistance, lipid abnormalities, and muscular metabolic dysfunction commonly observed in this syndrome.

This study has several limitations. First, its cross-sectional design precludes determining whether alterations in MOTS-c levels are a cause or a consequence of the metabolic disturbances observed in PCOS. Mitochondrial involvement was assessed using a single mitochondrial-derived peptide (MOTS-c) and therefore does not comprehensively reflect overall mitochondrial function. In addition, the sample size, particularly for muscle biopsies, was relatively small, which may limit the generalizability of the findings. Moreover, muscle MOTS-c assessment was restricted to expression analysis, without accompanying functional measurements, while dietary intake and structured physical activity were not controlled, both of which may influence mitochondrial peptide levels. Although multivariable analyses adjusting for age and BMI were performed, residual confounding by unmeasured factors cannot be excluded. Finally, the quantification of very low–molecular-weight peptides such as MOTS-c by Western blotting may be affected by technical factors including transfer efficiency and membrane retention, which should be considered when interpreting these findings. In addition, MOTS-c detection by Western blot is further complicated by reported variability in its apparent molecular weight across different tissues and experimental conditions, which should be considered when interpreting band localization.

In this study, we found that women with PCOS exhibit significantly reduced MOTS-c levels in both serum and skeletal muscle, suggesting that impaired mitochondrial peptide signaling may be associated with the metabolic phenotype of the syndrome. Further research is needed to elucidate the mechanistic role of MOTS-c in the pathophysiology of PCOS and its associated metabolic disturbances. Interventional studies involving exercise and dietary modification may help determine whether MOTS-c levels are modifiable and clinically relevant. Moreover, assessing a broader panel of mitochondrial-derived peptides in larger, longitudinal cohorts could help define their causal relationships and their potential utility as biomarkers or therapeutic targets in PCOS.

## Supplementary Information

Below is the link to the electronic supplementary material.

Supplementary Material 1

## Data Availability

The datasets generated and analyzed during the current study are available from the corresponding author upon reasonable request.
