# Correction: Reduced serum and skeletal muscle MOTS c levels in women with polycystic ovary syndrome are associated with mitochondrial dysfunction

**DOI:** 10.1038/s41598-026-70401-z

**Published:** 2026-09-09

**Authors:** Irem Sonmezoglu Kutuk, Senay Akin, Haydar Demirel, Sezcan Mumusoglu, Turkmen Ciftci, Bulent Okan Yildiz

**Affiliations:** 1 Department of Internal Medicine, Hacettepe University School of Medicine, Ankara, Turkey; 2 Division of Exercise and Sport Physiology, Faculty of Sport Sciences, Hacettepe University, Ankara, Turkey; 3 Center for Sport Sciences, Near East University, Nicosia, Cyprus; 4 Department of Obstetrics and Gynecology, Hacettepe University School of Medicine, Ankara, Turkey; 5 Department of Radiology, Hacettepe University School of Medicine, Ankara, Turkey; 6 Division of Endocrinology and Metabolism, Hacettepe University School of Medicine, Ankara, Turkey; 7 Division of Endocrinology and Metabolism, Department of Internal Medicine, Hacettepe University School of Medicine, Hacettepe, Ankara, 06100 Turkey

Correction to: *Scientific Reports* https://doi.org/10.1038/s41598-026-39687-x, published online 12 February 2026

The original version of this Article contained an error in the Materials and methods section, under subheading ‘Serum MOTS-c measurements’, where data and values of the ELISA kit were stated incorrectly. As a result,

“Serum MOTS-c concentrations were quantified using a commercially available enzyme-linked immunosorbent assay (ELISA) kit (Abbexa Cat# abx258343, RRID: AB_3717946), according to the manufacturer’s instructions. The intra-assay and inter-assay coefficients of variation were < 10% and < 12%, respectively, demonstrating high analytical reliability. According to the manufacturer’s specifications, the lower limit of detection of the assay was < 90.9 pg/mL.”

now reads:

“Serum MOTS-c concentrations were quantified using a commercially available enzyme-linked immunosorbent assay (ELISA) kit (BT Lab, Cat# E6880Hu), according to the manufacturer's instructions. According to the manufacturer's specifications, the sensitivity of the assay was 4.08 pg/mL, with a detection range of 7–1500 pg/mL.”

The original Article has been corrected.

