# Supplementary material for: Reduced serum and skeletal muscle MOTS c levels in women with polycystic ovary syndrome are associated with mitochondrial dysfunction
Source: Sci Rep. 2026 Feb 12;16:8593. doi: 10.1038/s41598-026-39687-x (PMC12976357; doi:10.1038/s41598-026-39687-x)
Supplement: Supplementary file 1 — Supplementary Material 1 [file 41598_2026_39687_MOESM1_ESM.docx]

| **Table S1. Comparison of baseline demographic and metabolic characteristics between the biopsy subgroup and the study cohort** | | | | | | |
| --- | --- | --- | --- | --- | --- | --- |
|  | **PCOS** | | | **Control** | | |
| Variable | Biopsy group (n=6) | Serum group (n=34) | p value | Biopsy group (n=6) | Serum group (n=34) | p value |
| Age | 23 (±1.8) | 21.6 (±2.4) | 0.186 | 23.2 (±1.7) | 22.4 (±1.6) | 0.274 |
| BMI (kg/m^2^) | 24.2 (22.7-25.6) | 24.4 (21.2-28.5) | 0.762 | 23.4 (20-25.3) | 22.7 (20.4-24.4) | 0.705 |
| Total testosterone (ng/dL) | 55.3 (40.2-60) | 54.2 (40-64.3) | 0.850 | 26.3 (23.5-30.2) | 26.2 (20.7-33) | 0.970 |
| SHBG (nmol/l) | 35.5 (25-50.1) | 46 (29.3-66.3) | 0.289 | 52.8 (46.5-79.8) | 52.6 (41.2-72.6) | 0.677 |
| FAI | 4.8 (3.5-5.6) | 3.5 (2.8-5.2) | 0.240 | 1.6 (1-2.1) | 1.7 (1.2-2.4) | 0.570 |
| Fasting glucose (mg/dL) | 82.2 (±4.6) | 85.4 (±9.4) | 0.423 | 86.2 (±7) | 83.3 (±5.1) | 0.235 |
| Fasting insulin (μIU/mL) | 7.1 (5-8.6) | 7.0 (5.6-11) | 0.520 | 4.1 (2.5-9.3) | 6.6 (5.2-8.5) | 0.289 |
| 2-hour glucose (mg/dL) | 80 (±9.8) | 91.5 (±19.0) | 0.159 | 82.7 (±21.8) | 82.7 (±13.5) | 0.998 |
| 2-hour insulin (mg/dL) | 27.3 (20.1-40) | 35.2 (23.7-74.8) | 0.211 | 25.8 (14.8-28.5) | 25.1 (13.7-41.1) | 0.622 |
| Total cholesterol (mg/dL) | 173.5 (±36) | 181.5(±33.1) | 0.591 | 181 (±54.1) | 163.1 (±27.3) | 0.473 |
| HDL-C (mg/dL) | 57.5 (±6.1) | 60 (±12.9) | 0.512 | 58.8 (±9.9) | 56.1 (±7.1) | 0.419 |
| LDL-C (mg/dL) | 108.7 (±34.3) | 113.7 (±24.7) | 0.668 | 109.3 (±38) | 97.6 (±19.9) | 0.490 |
| Triglyceride (mg/dL) | 64 (49-86) | 75 (53-112) | 0.373 | 61 (41-68) | 54 (43-76) | 0.830 |
| Data are given as mean (SD) and median (IQR) where appropriate.  SHBG: Sex hormone binding globulin, FAI: free androgen index, HDL: High density lipoprotein cholesterol, LDL: Low density lipoprotein cholesterol. | | | | | | |

## **Table S2. Multivariable linear regression analysis for determinants of serum MOTS-c levels**

| **Predictor** | **Unstandardized B** | **Standardized β** | **95% CI for B** | **p value** |
| --- | --- | --- | --- | --- |
| Age (years) | 41.55 | 0.378 | 18.72 to 64.38 | 0.001 |
| BMI (kg/m²) | 1.94 | 0.035 | −9.76 to 13.63 | 0.742 |
| Total testosterone (ng/dL) | −2.91 | −0.225 | −5.58 to −0.24 | 0.033 |

**Model statistics:**
R = 0.455; R² = 0.207; Adjusted R² = 0.176; F = 6.62; p < 0.001

**Dependent variable:** Serum MOTS-c (pg/mL)

## **Table S3. Multivariable linear regression analysis for determinants of serum MOTS-c levels (lipid-adjusted model)**

| **Predictor** | **Unstandardized B** | **Standardized β** | **95% CI for B** | **p value** |
| --- | --- | --- | --- | --- |
| Age (years) | 44.57 | 0.405 | 21.57 to 67.57 | <0.001 |
| BMI (kg/m²) | −0.09 | −0.002 | −11.73 to 11.55 | 0.988 |
| Total cholesterol (mg/dL) | −1.48 | −0.223 | −2.86 to −0.11 | 0.034 |

**Model statistics:**
R = 0.448; R² = 0.200; Adjusted R² = 0.168; F = 6.27; p = 0.001

**Dependent variable:** Serum MOTS-c (pg/mL)

## **Table S4. Multivariable linear regression analysis including group status (PCOS vs control) for serum MOTS-c levels**

| **Predictor** | **Unstandardized B** | **Standardized β** | **95% CI for B** | **p value** |
| --- | --- | --- | --- | --- |
| Age (years) | 35.83 | 0.326 | 13.36 to 58.31 | 0.002 |
| BMI (kg/m²) | 4.72 | 0.085 | −6.81 to 16.25 | 0.417 |
| Group status (PCOS vs control) | 149.07 | 0.337 | 57.99 to 240.16 | 0.002 |

**Model statistics:**
R = 0.511; R² = 0.261; Adjusted R² = 0.232; F = 8.96; p < 0.001

## **Table S5. Multivariable linear regression analysis including physical activity (IPAQ) for serum MOTS-c levels**

| **Predictor** | **Unstandardized B** | **Standardized β** | **95% CI for B** | **p value** |
| --- | --- | --- | --- | --- |
| BMI (kg/m²) | 5.37 | 0.096 | −6.41 to 17.14 | 0.367 |
| Physical activity (IPAQ score) | 173.65 | 0.372 | 75.13 to 272.18 | 0.001 |

**Model statistics:**
R = 0.377; R² = 0.142; Adjusted R² = 0.120; F = 6.37; p = 0.003

**Dependent variable:** Serum MOTS-c (pg/mL)

**
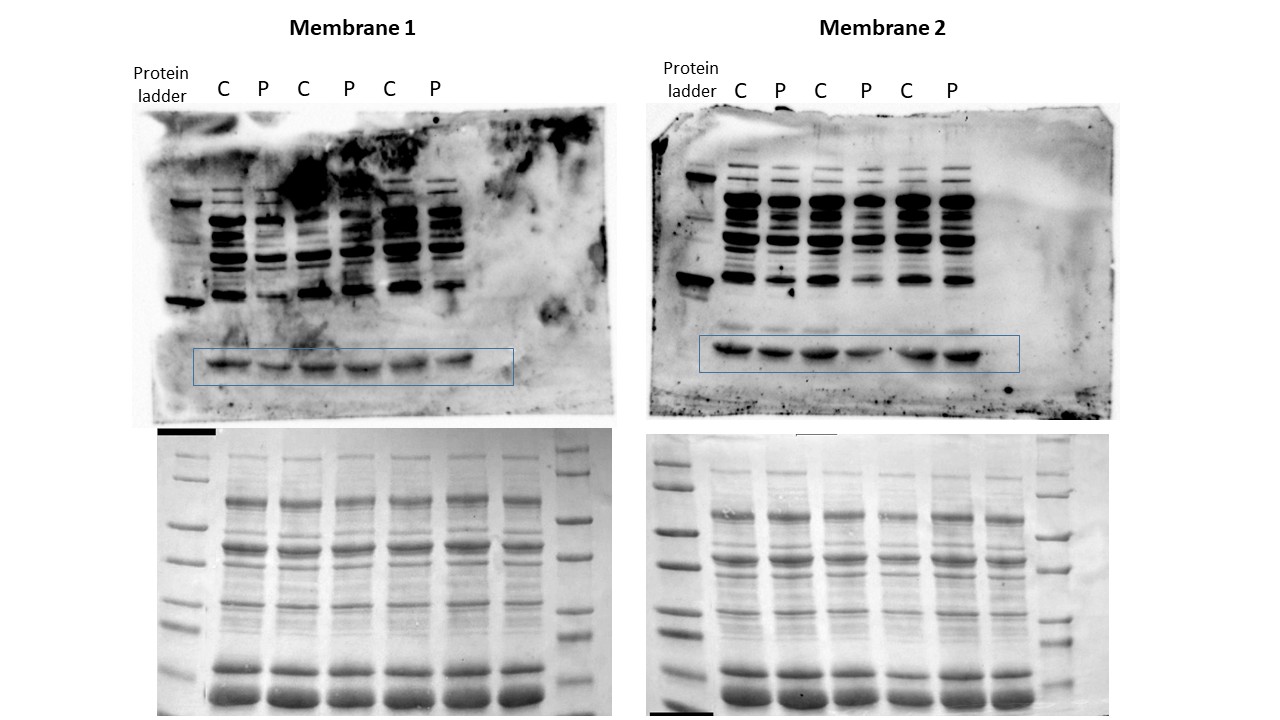
**

**Supplementary Figure S3. Uncropped Western blot membranes.**
Uncropped images of PVDF membranes corresponding to the Western blots shown in Figure 3, with membrane edges and molecular weight markers visible. Boxes indicate the regions presented in the main figures. Membranes were cut prior to antibody incubation to permit probing of proteins at different molecular weights. Images from all biological replicates are shown.
